# Sex steroids and glucocorticoid ratios in Iberian lynx hair

**DOI:** 10.1093/conphys/coaa075

**Published:** 2020-08-17

**Authors:** Alexandre Azevedo, Jella Wauters, Clemens Kirschbaum, Rodrigo Serra, António Rivas, Katarina Jewgenow

**Affiliations:** 1 Department Reproduction Biology, Leibniz Institute for Zoo and Wildlife Research, Alfred-Kowalke-Str.17, D-10315 Berlin, Germany; 2 Instituto de Ciências Biomédicas Abel Salazar, R. Jorge Viterbo Ferreira 228, 4050-313 Porto, Portugal; 3 Faculty of Psychology, Department of Biopsychology, Technical University of Dresden, Helmholtzstraße 10, D-01069 Dresden, Germany; 4 National Breeding Breeding Center for Iberian Lynxes, 8375-082 Messines, Portugal; 5 El Acebuche Iberian Lynx Captive Breeding Centre-OAPN, Doñana National Park, Matalascañas, 21760 Huelva, Spain

**Keywords:** Cortisol, cortisone, EIA, hair, HPLC, LC-MS/MS

## Abstract

Knowledge on species’ reproductive biology is a fundamental pre-requisite of every conservation effort, but is often lacking. Sex steroids can provide valuable information for the assessment of reproductive success, whereas glucocorticoids are used to assess adrenocortical activity and stress-related bodily adaption. However, due to their perilous condition, access to animals is often difficult, which makes hormone measurement in non-invasively collected hair samples an attractive option. We determined cortisol, cortisone, corticosterone, testosterone and progesterone in Iberian lynx hair using enzyme immunoassay (EIA). Cross-validation was performed with high-performance liquid chromatography (HPLC) and high-performance liquid chromatography coupled to tandem mass spectrometry (HPLC-MS/MS). Finally, we statistically evaluated the variations of sex steroids and glucocorticoids according to age, sex, origin, behavior and management. All steroids except corticosterone were detectable in Iberian lynx hair. Hair progesterone measured by EIA was overestimated by cross-reaction with 5α-dihydroprogesterone, a biologically active gestagene, and was highly correlated with HPLC-MS/MS results. Progesterone was higher in adult females compared to all other age-sex groups. Cortisol measured by EIA was overestimated due to antibody cross-reactivity with cortisone and was correlated to the sum of HPLC-MS/MS measurements for cortisol and cortisone. Cortisol was higher in females than in males measured by HPLC-MS/MS, but the EIA results were confounded by the lack of specificity. When using cortisol-cortisone and cortisol-dihydroepiandrosterone ratios, differences were noted between wild-caught and captive-bred lynxes. Additionally, longitudinal EIA measurements of an Iberian lynx after a wildfire showed an inversion of the cortisol-cortisone ratio that later subsided. These results validate the use of hair progesterone measurement for Iberian lynx reproductive monitoring and add to the growing evidence supporting the need for a more comprehensive approach to hair steroid measurement that accounts for local interconversion and co-regulation mechanisms.

## Introduction

Conserving and restoring populations of wild species has become a global necessity in the attempt to slow down current extinction rates (Hoffmann *et al*., 2010; Pimm *et al*., 2014). Common approaches involve interventions in the wild, coupled to captive breeding and management of ‘rescue net’ populations for reintroduction (Ebenhard, 1995; Mallinson, 1995). In many cases, the biology of the targeted species is poorly known, and knowledge needs to be gathered as part of the initial establishment of conservation programs. Questions related to species’ reproductive biology and physiology need to be answered promptly in order to ensure health, survival and reproduction in both captive and wild populations. Unfortunately, for many species this information is still lacking (Wildt *et al*., 2010). Measurement of steroid sex hormones provides a valuable tool for understanding the reproductive biology and ecology of wild species, thereby supporting development of assisted reproductive techniques (ARTs) (Wildt and Wemmer, 1999; Schwarzenberger, 2007; Comizzoli *et al*., 2009; Jewgenow *et al*., 2017). Similarly, the measurement of glucocorticoids has become accepted for the assessment of behavior, stress and allostatic load in captive and wild populations (Möstl and Palme, 2002; Sheriff *et al*., 2011; MacDougall-Shackleton *et al*., 2019). Steroid hormone measurements have traditionally been performed using enzyme immunoassays (EIAs), which remain the most viable option for wide-scale daily application in field conservation, despite the availability of more specific yet more expensive and skill-demanding techniques such as (ultra) high-performance liquid chromatography ((U)HPLC) facultatively coupled to liquid chromatography-mass spectrometry (LC-MS).

Animal populations under conservation management are often in perilous conditions, with very few specimens remaining and low genetic diversity. Because of the risks involved in capture, sampling procedures should be reduced to the minimum, complicating the use of matrices that need to be collected invasively and/or repeatedly, like blood serum or plasma. These limitations have been circumvented by measuring hormones and their metabolites in feces, urine and hair (Sheriff *et al*., 2011). Hair is thought to reflect the longest time period of circulating hormone levels in a single measurement, is easy to store for long periods and does not require special shipping conditions. It has unsurprisingly become popular for stress research in wildlife species over the past 2 decades (Meyer and Novak, 2012; Russell *et al*., 2012; Stalder and Kirschbaum, 2012). In the field of reproductive biology, measurement of hormones in hair has not been as popular, with validation studies performed in only a few species of wildlife (Koren *et al*., 2019).

Several authors have alerted to the uncertainties that should be addressed before applying EIA to measure steroids and their metabolites in feces (Touma and Palme, 2005; Pribbenow *et al*., 2017; Palme, 2019) and in hair (Koren *et al*., 2019). Currently, uncertainties persist on how hormones are incorporated into the hair shaft and how much local production of steroids influences the results (Ito *et al*., 2004; Slominski *et al*., 2007; Sharpley *et al*., 2009; Keckeis *et al*., 2012; Salaberger *et al*., 2016). Furthermore, assays used without validation can confound steroid measurements due to cross-reaction with unintended compounds (Jewgenow *et al*., 2020). Additionally, biological validation is necessary to understand which time period of circulating steroid levels is reflected in hair samples (Kalliokoski *et al*., 2019). Nevertheless, based on the response to adrenocorticotropic hormone (ACTH) stimulation tests and correlation with the values of fecal, blood and salivary glucocorticoids, it is currently well established that hair glucocorticoids (hGCs) reflect adrenocortical activity (Kalliokoski *et al*., 2019). However, while hormonal variations caused by gender or reproductive cycles provide opportunities for validation, the number of studies focusing on sex steroid measurement in hair is so far limited in wild (Koren *et al*., 2006; Terwissen *et al*., 2014; Bryan *et al*., 2015, 2014; Cattet *et al*., 2017) and domestic species (Gleixner and Meyer, 1997; Terwissen *et al*., 2014; Tallo-Parra et al., 2018; Bergamin *et al*., 2019).

At the turn of the century, the Iberian lynx (*Lynx pardinus*) was the most endangered felid on Earth, with less than 200 animals remaining (Guzmán *et al*., 2004). Extensive conservation efforts have led to a population recovery over the past 2 decades, allowing the species to be downgraded from ‘critically endangered’ to ‘endangered’ by the International Union for Conservation of Nature (IUCN). Despite the successful genetic management of captive and wild populations (Kleinman-Ruiz *et al*., 2019), it remains threatened by the lowest genome-wide genetic diversity reported so far in any species (Abascal *et al*., 2016). Wild felids often have poor reproductive success in captivity (Andrews *et al*., 2019). Abortions, premature birth and infant mortality have been challenging problems in the Iberian lynx captive breeding program, leaving ART as a last resort to salvage the genetic diversity of founders that did not reproduce within their lifetime (Jewgenow *et al*., 2017). These techniques are also expected to play a role in improving meta-population management, by allowing gene flow between the unconnected populations without the movement of animals (Swanson, 2006). Consequently, cost-effective and non-invasive tools for monitoring the reproductive cycles hold great potential for pinpointing the adequate timing of reproductive management interventions and for assessing reproductive success, both in captivity and in the wild. Similarly, the non-invasive measurement of glucocorticoids offers a promising tool for the measurement of chronic stress in the captive Iberian lynx population or of reintroduced animals adapting to the wild. Additionally, glucocorticoid levels may serve as an indicator of allostatic load in free-ranging populations. In this study, we therefore evaluated the potential of cortisol, cortisone, corticosterone, testosterone and progesterone determination in Iberian lynx hair using EIA. Each assay was validated through identification of the immuno-reactive components in the hair extracts after HPLC separation. Additional validation regarding the specificity of the EIA results was pursued in a subset of samples applying gold standard high-performance liquid chromatography coupled to mass spectrometry (HPLC-MS/MS). Once methods were validated, we evaluated the variations in concentrations of sex steroids, glucocorticoids and selected hormone ratios according to age, sex, origin and behavior and management variables. Finally, we presented an example of longitudinal variation of hGC measurements in an Iberian lynx after an escape-recapture event caused by a wildfire.

## Materials and methods

All chemical reagents were purchased from Sigma–Aldrich (Taufkirchen, Germany) unless stated otherwise and were of the highest purity available.

### Sample collection

Samples from Iberian lynx were obtained during routine health checks within the Iberian lynx conservation breeding program in Spain and Portugal. While all animals were held in captivity, some had been born in the captive-breeding program (captive-bred) while others had been brought into the breeding program from the wild as founders (wild-caught). Animals were captured for health checks with cage traps that they entered voluntarily after a period of habituation. Hair samples were collected opportunistically from the standard sites where hair was clipped for venipuncture while the animals underwent routine health checks under anesthesia. If not indicated otherwise, the hair was clipped from the inner surface of hind legs. For the comparison of hormone levels in proximal and distal segments of hair samples, hair was clipped first at 0.5 cm length to obtain the distal segment, and the remainder was clipped as close to the skin as possible to obtain the proximal segment of 0.5 cm length.

### Steroid extraction from hair

Previously, two methods were reported for the extraction of steroids in hair for glucocorticoid analysis (Jewgenow *et al*., 2020). The first method describes extracts for glucocorticoid quantification by immunoassays (pulverized method), while the second method was adapted from Carlitz *et al*. (2016) and characterized by the extraction of whole hair (whole hair method) prior to glucocorticoid identification by HPLC-MS/MS. We screened the applicability of both methods for steroid (glucocorticoids, progesterone and testosterone) analysis in lynx hair by monitoring the repeatability of the extraction after quantification by EIA. In a first experiment, we examined the repeatability of glucocorticoid, progesterone and testosterone quantification, expressed by the coefficient of variation (CV, %), after comparing pulverized and whole hair extractions in five male and five female individuals, with two replicates per method for each individual.

Based on the results, and taking into account the complexity of the lynx hair samples, the experiment was repeated after including an additional sample preparation step for both methods, which comprised cutting the available hairs in 5 mm pieces followed by mixing prior to weighing of 20 mg hair fragments for further extraction. For the whole hair extraction, the method was adapted to Mastromonaco *et al*. (2014), an established method for steroid extraction for hair in wildlife. The main differences compared to the original method were the fraction of MeOH (80% versus 100%), the shaking time (24 h versus 18 h) and the shaking temperature (room temperature compared to 45 C). In this follow-up experiment, hair subsamples were subjected to both extraction methods with duplicate analysis included for the adapted whole hair method (three males and three females) and triplicate analysis for the pulverized hair method (two males and three females). The final extraction method selected for sample processing is described below.

Hair was cut into 5 mm pieces, mixed and an aliquot of 20 mg separated and washed twice with 2 ml of 90% methanol by vortexing for 5–10 s to remove surface contamination. Thereafter, the samples were dried for 1 h at 70 C and aliquots (~10 mg) from selected Iberian lynx (*n* = 93) were taken and milled to a fine powder with ceramic beads in a tissue homogenizer as described before (Azevedo *et al*., 2019). Then 400 μl of 90% methanol were added to the powder and shaken at room temperature for 30 min. Following centrifugation (3 min, 1000 × g), the supernatant was collected and transferred into a new tube, diluted 1:2 with water and kept frozen until EIA analysis and the preparation of HPLC immunograms. From hair samples of 12 lynxes, additional extracts were produced for HPLC-MS/MS analyses (see below) according to the same protocol.

### Steroid analyses by EIA

An in-house cortisol immunoassay was used based on polyclonal antibodies (rabbit) directed against cortisol-3-CMO-BSA and the corresponding 3-CMO-peroxidases label as previously described (Ludwig *et al*., 2013). The antibody cross-reactivities of the cortisol-3-CMO assay were as follows: cortisol, 100%; cortisone, 19.5%; corticosterone, 6.3%; desoxycorticosterone, 0.1%; progesterone, 0.1%; estradiol, 0.1%; testosterone, 0.1%.

Cortisone was determined by a commercial EIA (Arbor Assays DetectX® Kit K017, Arbor Assays, An Arbor, USA). To comply with the manufacturer’s guidelines, a 75–150 μl aliquot of each sample extract was freeze dried and resolved in 150 μl assay buffer. The EIA measurements were performed in duplicates as indicated for the kit. According to the providers, the cross-reactivities were as follows: cortisone, 100%; 5α-dihydrocortisone, 31.7%; prednisone, 9.0%; 5ß-dihydrocortisone, 4.4%; 11-dehydrocorticosterone, 0.62%; 20α-dihydrocortisone, 0.26%; < 0.1% for 11α-hydroxycorticosterone, 20ß-dihydrocortisone, corticosterone, cortisol, dexamethasone, estradiol and progesterone.

Progesterone (P4) analyses were carried out with an in-house microtitre plate EIA as described earlier (Dehnhard *et al*., 2008) using a commercial P4 antibody (Sigma P1922, raised in rats to progesterone) and 4-pregnen-3,20-dione-3-CMO-peroxidase label. The cross-reactivities to other steroids were as follows: 4-pregnen-3,20-dione (progesterone), 100%; 5α-pregnan-3,20-dione (5α-DHP), 76.8%; 5α-pregnan-3ß-ol-20-one (5a), 64.2%; 5-pregnen-3ß-ol-20-one, 12%; 4-pregnen-3αol-20-one, 4.2%; < 0.1% for 5ß-pregnan-3α,20αdiol, 4-pregnen-20α-ol-3-one, 5ß-pregnan-3α-ol-20-one, 5a-pregnan-20α-ol-3-one, 5α-pregnan-3α,20α-diol, 5a-pregnan-3ß,20α-diol, testosterone, estradiol and cortisol.

Testosterone was analyzed with an in-house EIA, using polyclonal antibodies (rabbit) against testosterone-3-CMO-BSA and the corresponding 3-CMO-peroxidase as label. The cross-reactivities were as follows: testosterone, 100%; dihydrotestosterone (DHT), 53.85%; 4-androsten-3ß,17ß-diol, 4.6%; 19-nortestosterone, 2.3%; < 0,01% for 11ß-hydroxyetiocholanolone, 11-oxo-etiocholanolone, cortisol, corticosterone, 5α-androstan-17-one, androstendione, androsterone, 5α-androsterone, dihydroepiandrosterone (DHEA), testosterone-glucoronide and epiandrosterone.

The principle of the in-house EIA procedure has been described in detail (Finkenwirth *et al*., 2010). All EIA measurements were conducted in duplicate and results were expressed as pg/mg hair weight. Serial dilutions of hair extracts showed parallelism to the standard, with no significant difference in slopes (*P* > 0.05). The inter- and intra-assay coefficients of variation (CV) for the EIA as well as the (linear) detection range are presented in Table S1 of the Supplementary Materials.

### High-performance liquid chromatography coupled to mass spectrometry

Hair extracts from 12 selected Iberian lynxes (six males, six females) were adjusted to an absolute content of about 150 pg cortisol. To achieve this, cortisol concentration of extracts were determined by cortisol-3-CMO-EIA, and the required volume of each sample was determined according to the mean cortisol concentration (see Supplementary Table S2). For Iberian lynx, 200 μl (~5 mg hair) of each extract were transferred to 1.5 ml reaction tubes, dried down for 90 min at 60 C under a constant stream of nitrogen and dissolved in 225 μl 50% methanol (LC-MS grade). The samples were sent to the Faculty of Psychology, Technische Universität Dresden, Germany, for HPLC-MS/MS analyses, based on a Shimadzu LC-20AD HPLC unit, a Shimadzu SIL-20AC autosampler and a Shimadzu CTO-20AC column temperature oven (Shimadzu, Canby, OR, USA) coupled to an AB Sciex API 5000 Turbo-ion-spray® triple quadrupole tandem mass spectrometer equipped with spheric pressure chemical ionization (APCI). Details on the liquid chromatography methodology and mass spectrometric conditions are described in Carlitz *et al*. (2016) and Gao *et al*. (2013).

### High-performance liquid chromatography

HPLC was performed as described before (Jewgenow *et al*., 2020) and aimed to characterize the compounds detected by the respective EIA. From the hair extracts of 93 lynxes, aliquots of 460 μl were taken corresponding to ~530 mg of hairs and pooled before purification on Octadecyl C18 columns (0.5 ml, J.T. Baker, BAKERBOND SPE™ 7020-01) as previously described (Azevedo *et al*., 2019). Eluates were evaporated in a heater at 55 C under nitrogen and dissolved in 200 μl of 40% methanol. About 130–150 μl of the re-suspended volume was injected on a reverse-phase Ultrasep ES100/RP – 18/6 μm HPLC column (4 × 250 mm, Sepserv, Berlin) and separation was achieved by using a methanol + water mixture with the following gradient: 60% methanol during 5 min, 60–90% methanol during 10 min, 90–100% methanol during another 10 min. The flow rate was 1 ml/min. Fractions of 0.33 ml were collected at 20 s intervals over a period of 25 min. All fractions were lyophilized and re-suspended in 200 μl 40% methanol before 20 μl aliquots were analyzed by EIA. The elution positions of native cortisone, cortisol, corticosterone, 11-hydroxyetiocholanolone, testosterone (T), progesterone (P4), 5α-pregnan-3,20-dione (5α-DHP) and 5α -pregnan-3α,20α-diol (5a) on this column had been previously determined in separate HPLC runs.

### Statistical methods

All statistical analyses were conducted in R version 3.5.1 (R Core Team, 2017). The association between paired values of hormone EIA measurements from proximal and distal segments of hair samples was tested using Pearson’s correlation, following the assessment of scatterplots for linear covariation and Shapiro–Wilk normality tests and quantile–quantile plots for normality. In the single case of non-normality (cortisone), Spearman’s rank-based correlation was used. For comparisons of the variation of each hormone EIA according to age class (animals between 6 months and 2 years of age were considered juveniles, while animals older than 2 years of age were considered adults) and sex, a two-way analysis of variance with type III sum of squares for unbalanced sample sizes was conducted using the EIA results as dependent variables and age, sex and the interaction between age and sex as independent variables. Model residuals were checked for normality and homoscedasticity by visual inspection of residual plots and Shapiro–Wilk normality tests. Homogeneity of variances was checked by visual inspection of plots and Levene’s test. *Post hoc* between-group tests were performed with Tukey–Kramer adjusted comparisons, using the multcomp package (Hothorn *et al*., 2008). For the cortisol-cortisone ratio (Sollberger and Ehlert, 2016), a Kruskal–Wallis non-parametric test using age-sex group as the independent variable was used, followed by Dunn’s *post hoc* test with Bonferroni correction, using the dunn.test package (Dinno, 2017). Mann–Whitney U-tests were used for the comparison of glucocorticoid EIA values and the cortisol-cortisone ratio (Sollberger and Ehlert, 2016) between captive-bred and wild-caught lynxes. For an analysis of the variation of hGC levels measured by cortisol-3CMO-EIA in relation to behavioral (mean frequency of repetitive behavior in the last month and the last year) and management variables (breeding center, enclosure area, days since last capture, number of lifetime captures, days since last transfer between institutions, number of lifetime transfers between institutions, days since last enclosure change and number of lifetime enclosure changes) available for a subset of females (*n* = 19), non-parametric Kruskal–Wallis, Mann–Whitney U-tests and Spearman’s rank correlation tests were used. Comparisons of HPLC-MS/MS hormone measurements, and their ratios, between Iberian lynxes of different age, sex and origin (wild caught versus captive bred) were performed using non-parametric tests due to the small sample size (*n* = 12) and the non-normal distribution of complex variables such as ratios (Sollberger and Ehlert, 2016). Mann–Whitney U-tests were used for sex and origin, and Spearman’s rank correlation was used for age.

## Results

### Steroid extraction from hair

Both the pulverized and whole hair methods initially showed a poor repeatability of steroid extraction from hair samples, significantly underperforming compared to the generally accepted limits of up to 15% (20% for small levels) of variation between duplicate or triplicate extractions from the same hair sample. Consequently, an extra homogenizing step that consisted in cutting and mixing hair samples prior to sample weighing and extraction was included in the protocol for both methods. This extra preparatory step improved the repeatability to within the acceptable limits (Supplementary Table S3) and was therefore included in our standard protocol for all subsequent analyses.

No significant concentration differences were observed between the pulverized and whole hair methods for all steroids in combined data of males and females. Nevertheless, the pulverized hair method consistently resulted in higher concentrations for progesterone in males, and in both males and females for testosterone, when compared to the whole hair method. Measured steroid concentrations with both the pulverized and whole hair methods, including gender comparison, are presented in the Supplementary Materials (Table S3).

Due to the best observed repeatability in the samples treated with the pulverized hair method, particularly for progesterone, this method was selected for extraction of the study samples.

### Determination of steroid content in hair samples using EIA

**Figure 1:**
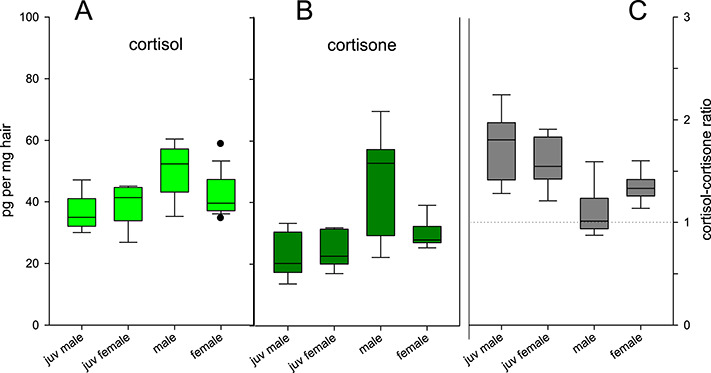
Iberian lynx hGCs (EIA) by age-sex group. Measurements of cortisol-3CMO-EIA (**A**) and cortisone-EIA (**B**) in pg/mg and calculated cortisol-to-cortisone ratio (**C**) based on EIA results, of hair from juvenile (*n* = 9) and adult male (*n* = 10), as well as juvenile (*n* = 9) and adult female (*n* = 13) Iberian lynxes

**Figure 2:**
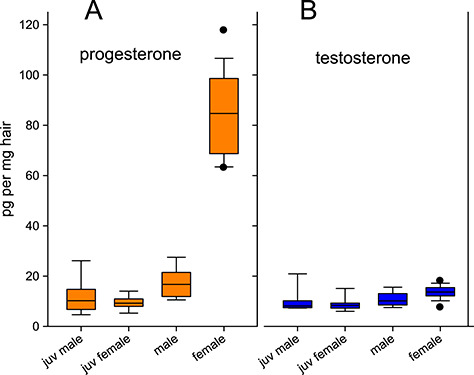
Sex steroids in Iberian lynx hair (EIA) by age-sex group. Measurements of progesterone-3CMO-EIA (**A**) and testosterone-3CMO-EIA (**B**) in pg/mg in hair from juvenile (*n* = 9) and adult male (*n* = 10), as well as juvenile (*n* = 9) and adult female (*n* = 13) Iberian lynxes

All of the targeted steroids were measureable in Iberian lynx hair samples using EIA (Supplementary Table S4). With the exception of testosterone, all exhibited differences when comparing different age and sex groups (Figs 1 and 2). In the case of the cortisol-3CMO-EIA, sex had a significant effect on hGC measurements (F_(1, 33)_ = 4.474, *P* = 0.004). *Post hoc* Tukey–Kramer pairwise comparisons showed that juvenile females had lower hGC levels measured by the cortisol-3CMO-EIA than adult males (Δ = −11.8 pg/g, 95% CI [−1.49, −11.81], *P* = 0.02), as did juvenile males (Δ = −13.4 pg/g, 95% CI [−2.83, −24.06], *P* = 0.01). The values measured with the commercial cortisone EIA were significantly influenced by sex (F_(1, 25)_ = 8.499, *P* = 0.007) and the interaction between age and sex (F_(1, 25)_ = 5.446, *P* = 0.027). *Post hoc* pairwise comparisons revealed higher cortisone measurements in adult males compared to adult females (Δ = 15.6 pg/mg, 95% CI [0.88, 30.35], *P* = 0.03), juvenile females (Δ = 21.4 pg/g, 95% CI [6.38, 36.49], *P* < 0.001) and juvenile males (Δ = −22.9 pg/g, 95% CI [−7.40, −38.33], *P* = 0.002). For progesterone EIA measurements, there were statistically significant effects of the age-sex interaction (F_(1, 33)_ = 81.66, *P* < 0.001), as well as age (F_(1, 33)_ = 220.07, *P* < 0.001) and sex (F_(1, 33)_ = 161.82, *P* < 0.001), with females presenting higher progesterone levels than males (Δ = −67.0 pg/mg, 95% CI [−81.36, −52.83], *P* < 0.001), juvenile males (Δ = −73.1 pg/mg, 95% CI [−87.34, −58.80], *P* < 0.001) and juvenile females (Δ = −75.6 pg/mg, 95% CI [−89.38, −61.81], *P* < 0.001). A significant effect of age (F_(1, 33)_ = 6.6485, *P* = 0.015) on testosterone-EIA measurements was present, but *post hoc* pairwise comparisons revealed no significant differences between groups. When using the calculated cortisol-cortisone ratio, the results showed significant differences between age-sex groups (χ2 = 11.90, df = 3, *P* = 0.01). *Post hoc* Dunn’s test revealed significant differences between adult and juvenile males (*P* = 0.007) and adult males and juvenile females (*P* = 0.023).

**Figure 3:**
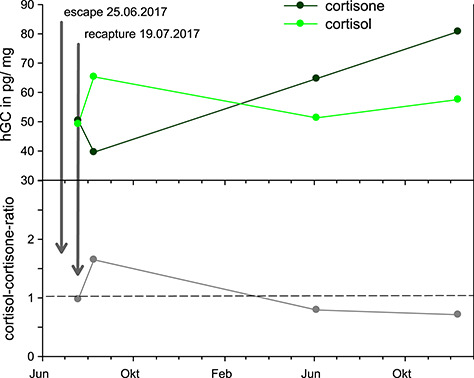
Inversion of cortisol-cortisone ratio in an Iberian lynx after a wildfire. Variation in cortisol-3CMO-EIA and cortisone-EIA measurements and their ratio in hair samples from an Iberian lynx named Fran, following a 24-day escape-recapture event caused by a massive wildfire that overcame the El Acebuche breeding center in 2017. Approximately 3 weeks after recapture, the relation between cortisol and cortisone had inverted, and 1 year later the inversion had been reversed

No significant difference was observed between EIA measurements of cortisol, cortisone and cortisol-cortisone ratio between wild-caught (*n* = 7) and captive-bred (*n* = 30) lynxes. Similarly, no effect of management and behavioral variables was found on hGCs measured by cortisol-3CMO-EIA, in the subset of females (*n* = 19) where these data were available (Supplementary Table S5). When comparing the EIA measurements between proximal and distal segments of the hair shafts (five males, five females), we found significant positive correlations for all the steroid hormones measured (Supplementary Table S6). The longitudinal follow-up of hGC levels in an Iberian lynx female following a 24-day escape-recapture event caused by a massive wildfire showed an increase in glucocorticoid levels measured by the cortisol-3CMO-EIA and a decrease in cortisone-EIA measurements ~3 weeks after recapture, resulting in an inversion in cortisol-cortisone ratio in relation to the recapture date (see Fig. 3). One year after recapture, cortisone-EIA measurements had increased and cortisol-3CMO-EIA measurements had decreased, thus reversing the inversion in cortisol-cortisone ratio that occurred in the 3 weeks post recapture.

### HPLC analyses of immunoreactive steroids

**Figure 4:**
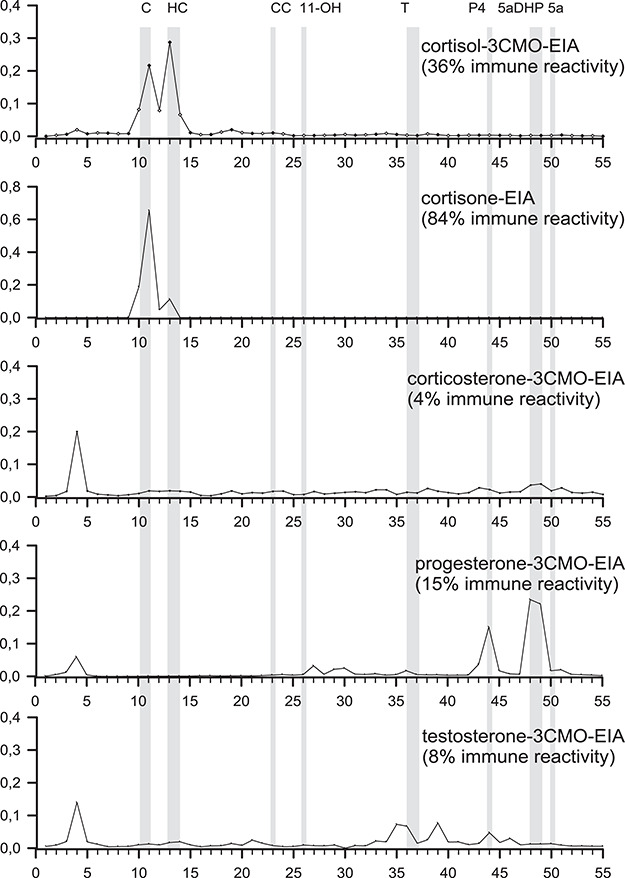
HPLC immunograms for EIA targeting steroid hormones in Iberian lynx hair. High performance liquid chromatography (reversed phase) separations of immunoreactive cortisol and progesterone metabolites in pooled hair samples from Iberian lynx *(Lynx pardinus*, *n* = 182, 900 mg hair). The obtained fractions were analyzed with a cortisol-3-CMO-EIA, a commercial cortisone-EIA, a corticosterone-3-CMO-EIA, a progesterone-3CMO-EIA and a testosterone-3CMO-EIA. The elution positions of reference standards are indicated by vertical lines, 10/11: C (cortisone), 13/14: HC (cortisol), 23: CC (corticosterone), 26: 11-OH (11-hydroxyetiocholanolone), 36/37: T (testosterone), 44–45: P4 (progesterone), 48–49: 5α-DHP (5α-pregnane-3,20-dione) 50: 5a (5α-pregnan-3ß-ol-20-one)

The HPLC immunograms (Fig. 4) show that the cortisol-3-CMO EIA reacted to both cortisol (fraction 13/14; 36%) and cortisone (fraction 10/11; 30%) with an overall immunoreactivity of 66%. Only marginal amounts of reactivity were detected throughout the remaining immunogram, indicating the EIAs specificity for these two hair glucocorticoids (therefore referred to as hGC). An even more specific HPLC-immunogram was obtained with the cortisone EIA, with only two peaks appearing at positions 10/11 (84%) and 13/14 (11%), which correspond to a total of 95% reactivity on a mostly non-reactive baseline.

In the case of the corticosterone-3-CMO-EIA, HPLC failed to detect any immunoreactivity in the positions corresponding to corticosterone (fractions 23). Peaks of cross-reactivity with unknown compounds were detected in fraction 4. The results are coincident with the very low or undetectable concentrations of testosterone and corticosterone, respectively, in the HPLC-MS/MS results for Iberian lynx hair.

In the case of the progesterone-3CMO-EIA, two peaks were identified in positions 44 and 48/49, corresponding to the elution positions of progesterone (15%) and 5α-pregnan-3,20-dione (5α-DHP; 46%), respectively. Other than a small 5% peak in fraction 3, there is little presence of cross-reactivity with known and unknown compounds, revealing that this EIA is adequate for the measurement of gestagens in Iberian lynx hair samples. The immunogram for the testosterone-3CMO-EIA revealed only 8% immunoreactivity in the position corresponding to testosterone (fraction 38).

### Determination of steroid content in hair samples using HPLC-MS/MS

**Figure 5:**
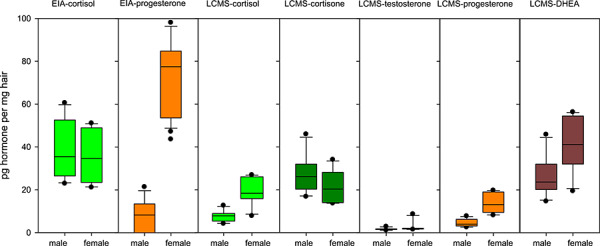
Steroid content in Iberian lynx hair measured by HPLC-MS/MS. Box-plots of steroid content in 12 Iberian lynx (six males, six females) determined by UHPLC-MS/MS. Values determined by cortisol-3-CMO-EIA and progesterone-3CMO-EIA in the same 12 samples were included for comparison. Sex differences are seen for progesterone and cortisol content in HPLC-MS/MS results. The difference between males and females appears overestimated in the progesterone-3CMO-EIA and attenuated in the cortisol-3CMO-EIA

We performed quantitative HPLC-MS/MS determinations of cortisol, cortisone, progesterone, testosterone and DHEA in a subset of 12 Iberian lynx samples (six males, six females) [LOD (limit of detection) = 0.32 pg/mg, LOQ (limit of quantification) = 1.05 pg/mg]. Cortisol and progesterone contents in these samples were also determined by the respective EIA to allow comparison (Fig. 5, Supplementary Table S7). The steroid recovered in highest concentration was DHEA, followed by cortisone and then cortisol. Sex steroids progesterone and testosterone were detected in lower concentrations, while corticosterone was not detectable.

The variation of HPLC-MS/MS steroid measurements and cortisol-to-cortisone and cortisol-to-DHEA ratios was analyzed according to sex (six female, six male), age (2 to 12 years) and origin (six wild caught, six captive bred). When comparing the results between sexes (Fig. 5), females presented significantly higher concentrations of progesterone and cortisol. The latter difference was also significant when evaluating sex differences in the cortisol-to-cortisone, but not the cortisol-to-DHEA ratio. No relation was found between age and any of the steroids measured. Comparison of HPLC-MS/MS measurements according to origin revealed a trend towards higher cortisol in wild-caught animals. This difference became highly significant when using cortisol-cortisone and cortisol-DHEA ratios (Fig. 5, Table 1).

**Table 1:** Comparison of HPLC-MS/MS hormone measurements according to origin and sex

	**Median ± inter-quartile range**		**Mann–Whitney U-test**	
**Variable**	**Captive bred** **(*n* = 6)**	**Wild caught** **(*n* = 6)**	**U**	***P*-value**
Cortisol (pg/mg)	8.09 ± 1.37	18.37 ± 8.62	6	0.065
Cortisone (pg/mg)	26.49 ± 7.18	16.50 ± 10.77	28	0.132
DHEA (pg/mg)	29.45 ± 19.14	35.06 ± 27.84	17	0.937
**Cortisol/cortisone (ratio)**	0.37 ± 0.13	0.98 ± 0.29	4	**0.026**
**Cortisol/DHEA (ratio)**	0.27 ± 0.01	0.48 ± 0.05	1	**0.004**
**Variable**	**Male** **(*n* = 6)**	**Female** **(*n* = 6)**	**U**	***P*-value**
**Cortisol (pg/mg)**	7.84 ± 2.95	18.37 ± 8.62	33	**0.015**
Cortisone (pg/mg)	26.16 ± 9.98	20.32 ± 12.68	11	0.309
DHEA (pg/mg)	23.68 ± 10.49	41.19 ± 18.42	27	0.180
**Progesterone (pg/mg)**	4.00 ± 2.72	13.15 ± 7.62	36	**0.002**
Testosterone (pg/mg)	1.66 ± 0.28	1.82 ± 0.29	26	0.240
**Cortisol/cortisone (ratio)**	0.28 ± 0.14	0.98 ± 0.29	34	**0.009**
Cortisol/DHEA (ratio)	0.28 ± 0.01	0.48 ± 0.05	30	0.065

HPLC-MS/MS hormone measurements according to origin (six captive born and six wild caught) and sex (six male and six female) in Iberian lynxes held in the captive breeding program. Results are presented as median ± inter-quartile range and Mann–Whitney U-test results for values of cortisol, cortisone, DHEA, progesterone, testosterone, cortisol-cortisone ratio and cortisol-DHEA ratio.

### Relation between HPLC-MS/MS and EIA measurements

We performed regression analysis between the results of cortisol-3CMO-EIA and progesterone-3CMO-EIA and the HPLC-MS/MS results for the same 12 hair samples. The analysis indicated a significant relation between the values obtained with the cortisol-3CMO-EIA and the HPLC-MS/MS values of cortisone (r = 0.84, *P* < 0.001) and cortisol + cortisone (named hGC) (r = 0.86, *P* < 0.001), but not with cortisol alone. In the case of the progesterone-3CMO-EIA, the results showed a significant correlation with the progesterone values measured by HPLC-MS/MS (r = 0.86, *P* = 0.018).

## Discussion

In this study, we analyzed Iberian lynx hair samples for cortisol, cortisone, corticosterone, DHEA, progesterone and testosterone, using a combination of EIA, HPLC and HPLC-MS/MS to validate a group of assays that are applicable in field conservation. Our analysis shows that besides cortisol, cortisone, DHEA, progesterone and testosterone are detectable in Iberian lynx hair. DHEA was the most abundant steroid, followed by cortisone, cortisol, progesterone and testosterone, respectively. Corticosterone was not detectable.

Sex steroids such as progesterone and testosterone are important indicators for Iberian lynx conservation due to their potential role in pregnancy diagnosis and fertility assessment, respectively. The lynx genus is a unique case among felids due to the persistence of *corpora lutea* and elevated systemic progesterone levels beyond the duration of pregnancy (Jewgenow *et al*., 2014). Additionally, variations in sex hormone concentrations in hair have been associated with social and nutritional stress in wolves (*Canis lupus*) and brown and black bears (*Ursus arctos, Ursus americanus*) (Bryan *et al*., 2015, 2014) offering potential as indicators of pressure due to environmental change and for monitoring of reintroduction success. Our results revealed substantial amounts of progesterone in Iberian lynx hair, measureable both by HPLC-MS/MS and with the progesterone-3CMO-EIA, while testosterone was present in only small amounts.

Characterization of the progesterone-3CMO-EIA immunoresponse using HPLC showed immunoreactivity mostly to progesterone and 5α-DHP. The measurement by EIA resulted in a 3-fold overestimation of progesterone in hair when compared to the HPLC-MS/MS results, demonstrating the necessity for validation before assay application. The overestimation for progesterone in hair based on the progesterone-3CMO-EIA is not surprising and easily explained. As mentioned earlier, HPLC-MS/MS offers the advantage of high sensitivity combined with high specificity, allowing the unequivocal identification and quantification of a single compound. The specificity of EIA, however, depends strongly on the cross-reactivity of the antibody. In this case, a high cross-reactivity was reported for 5α-DHP (76.8%) and other progestins. 5α-DHP is a biologically active 5α-reduced metabolite of plasma progesterone and precursor for other progestins. HPLC analysis (Fig. 4) showed a three times higher immunoreactivity for 5α-DHP (46%) compared to progesterone for the progesterone-3CMO-EIA, hence explaining the consistently higher levels measured with EIA compared to HPLC-MS/MS. Nevertheless, the progesterone-3CMO-EIA confirmed to be suitable for reliable analysis of progestins in lynx hair. Progestin levels measured by EIA showed significant differences between groups, with adult females presenting higher levels than other age-sex classes (Fig. 2).

A large difference in circulating progesterone levels between non-breeding females, juveniles and males is not commonly expected for feline species, which usually express an induced ovulation. Active *corpora lutea* are usually only found after mating with elevated progesterone tied to formation of *corpora lutea* and persisting throughout pregnancy (Verhage *et al*., 1976; Swanson *et al*., 1995). Accordingly, hair progesterone is increased during pregnancy in domestic cats in comparison to spayed females (Terwissen *et al*., 2014). In contrast to brown bears (Cattet *et al*., 2017), hair of adult non-pregnant cats contains progesterone levels comparable to pregnancy either indicating a former (recent) luteal activity or a prolonged storage of incorporated progesterone within the hair matrices. The possibility of prolonged stability of progesterone in hair is further supported by the high correlation we found between measurements in different hair segments (Supplementary Table S6). The difference between adult females, irrespective of their actual reproductive status, in comparison to males or juveniles was however expected for females of the lynx genus due to the above-mentioned presence of persistent corpora lutea (Jewgenow *et al*., 2014; Painer *et al*., 2014). In the lynx, elevated serum progesterone levels are found throughout the year, including inactive reproductive periods (Göritz *et al*., 2009). Previous measurements performed on hair from Canada lynx (*Lynx canadensis*) pelts also identified higher progesterone levels in adult females when compared to males, although with some overlap between classes (Terwissen *et al*., 2014). Importantly, the ranges of progesterone values in Iberian lynx females, with all samples collected during the anestrus period (November–December) did not overlap with those of males in any age group in this study.

Like many solitary felids, the Iberian lynx’s spatial organization functions as a land tenure system with little or no intra-sexual overlap in core areas of each home range (Ferreras *et al*., 1997). Therefore, with further refinement, our data suggest there is potential for identification of territorial adult Iberian lynx females in the field based on progesterone measurement in hair samples. Progesterone values in blood samples of Iberian lynx were 4.3 ng/ml (*n* = 19) and 17.0 ng/ml (*n* = 14) in non-pregnant and pregnant females, respectively (Göritz *et al*., 2009), indicating a 3-fold increase during pregnancy. As a result of opportunistic sampling, our study is limited by the absence of samples from the active reproductive period, but future studies with samples of captive lynxes covering a broad array of the reproductive cycle may characterize hair progesterone variation throughout the year. This study clearly indicates that hair progesterone is a promising indicator of sex and maturity in (female) lynxes. Additionally, understanding how progesterone varies in hair, especially during estrus, pregnancy and lactation or anestrus can potentially provide a non-invasive method for (retrospective) pregnancy diagnosis in the wild. This is especially relevant for estimating reproductive success in elusive species like lynxes.

In contrast to progesterone, hair testosterone is not a suitable indicator of sex since it was detected at very low levels in Iberian lynx hair, both by HPLC-MS/MS and EIA. The latter method yielded a 4-fold overestimation, most likely associated with cross-reacting androgens. The mean testosterone values detected by HPLC-MS/MS in adult Iberian lynx hair samples (2.36 ± 2.04 pg/mg, *n* = 12; no difference between sexes) were similar to those reported in the Canada lynx (3.35 ± 1.65 pg/mg, *n* = 45) based on EIA (Terwissen *et al*., 2014). The mean testosterone values determined with the testosterone-3CMO-EIA were between 5 pg/mg (in juvenile females) and 25 pg/mg (and juvenile males). The HPLC immunograms for our testosterone EIA revealed that besides a small percentage of testosterone (<10%), many unknown hair-borne substances contribute to the EIA values, making the results difficult to interpret. Previously, we clearly demonstrated that lack of antibody specificity may skew targeted hormone analysis and lead to erroneous results (Jewgenow *et al*., 2020). Nevertheless, in this study it is hard to evaluate the specificity of the assay’s antibody due to low testosterone levels in lynx hair as confirmed by HPLC-MS/MS. A higher level of testosterone in adult male lynx hair was however expected as outcome of this study. Serum testosterone concentration of Iberian lynx males (0.40 ± 0.22 ng/ml, *n* = 11, range 0.14–0.89 ng/ml) exceeds the levels of females and subadults (below limit of detection of EIA) by several orders (Jewgenow unpublished results, but see also Jewgenow *et al*. (2006) for Eurasian lynx). However, this difference is not reflected in the hair testosterone levels of our Iberian lynx samples or Canada lynx pelts (Terwissen *et al*., 2014). Indeed, sex differences in hair testosterone concentrations were undetectable in most studies in carnivorans (Bryan *et al*., 2014; Terwissen *et al*., 2014; Schell *et al*., 2017). The two exceptions were one study in the grey wolf (*Canis lupus*), where males present higher concentrations than females (Bryan *et al*., 2015) and one in brown bears (*Ursus arctos*), where females had higher testosterone levels than males (Cattet *et al*., 2017). One explanation might be that testosterone is not incorporated into the hair matrix at the same rate as other steroid hormones (or at all) and does not reflect the serum concentration at any time point of the male reproductive cycle. Another possibility is that it is incorporated as another steroid, as described by Kapoor *et al*. (2018) for cortisol and cortisone. Testosterone can be metabolized to estradiol by aromatase and to 5α-dihydrotestosterone (5α-DHT) by 5α-reductase enzymes. 5α-reductase expression has been demonstrated in the skin of several mammal species, where it regulates 5α-DHT effects on hair growth (Yamana *et al*., 2010; Azzouni *et al*., 2012; Robitaille and Langlois, 2020). The fact that the hair follicle is a target organ for testosterone and 5α-DHT could explain the need of a local regulation mechanism to protect follicle function from the potential influence of reproductive fluctuations of serum testosterone. Studies with radiolabeled testosterone could be useful in clarifying this hypothesis.

Regarding glucocorticoids, our HPLC-MS/MS results show overall higher concentrations of cortisone (24.82 ± 9.58 pg/mg) compared to cortisol (13.35 ± 7.80 pg/mg). Growing evidence supports taking cortisol-to-cortisone interconversion into account when studying hGC incorporation. Raúl *et al*. (2004) noted a higher concentration of cortisone in relation to cortisol in hair when compared to blood, and more recently, Kapoor *et al*. (2018) demonstrated that radiolabeled cortisol injected intravenously was incorporated into hair as cortisol, cortisone and unknown glucocorticoid metabolites. Two 11β-hydroxysteroid dehydrogenase (11β-HSD) isoenzymes are known to inter-covert cortisol (active glucocorticoid) and cortisone (inactive glucocorticoid) in several species (Tomlinson and Stewart, 2001). Isoform 11β-HSD-1 converts cortisone to cortisol and is expressed, among other tissues, in the human epidermis, while isoform 11β-HSD-2 converts cortisol to cortisone and is expressed in human sweat glands (Hirasawa *et al*., 1997; Tomlinson and Stewart, 2001). These isoenzymes regulate cortisol availability, enabling the necessary amount of cortisol in circulation and specific organs to ensure homeostasis, while protecting mineralocorticoid receptors from binding with excessive cortisol (Tomlinson and Stewart, 2001). Local cortisol-to-cortisone conversion by 11β-HSD-2 in eccrine sweat glands (Raul *et al*., 2004) and different rates of incorporation of cortisone due to its lower polarity (Raul *et al*., 2004; Kapoor *et al*., 2018) have been suggested to explain the high amount of cortisone present in hair. The absence of functional eccrine sweat glands in hairy skin of mammals (Montagna, 1967) makes the first possibility unlikely in the present case. Additionally, progesterone, a more polar hormone than cortisol and cortisone, showed higher correlation between values of proximal and distal hair segments (Supplementary Table S7), thus suggesting an easier diffusion along the hair shaft. While most studies of hair steroids in mammals quantified only cortisol (Koren *et al*., 2019), the higher amounts of cortisone and DHEA that we detected in Iberian lynx hair by HPLC-MS/MS, together with the results of radiolabel validation studies (Keckeis *et al*., 2012; Kapoor *et al*., 2018) make it unclear whether the use of an EIA targeting only cortisol is sufficiently informative as a proxy of chronic activation of the stress response in this species. In fact, when investigating the relation of management and behavioral variables on cortisol alone measured by EIA in 19 captive Iberian lynx females, we found no effect (Supplementary Table S5).

**Figure 6:**
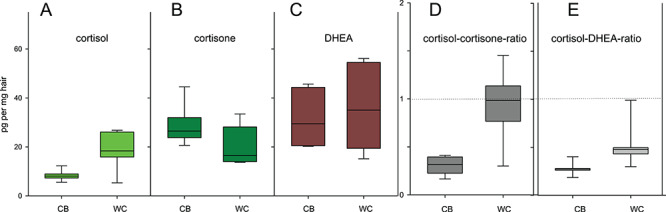
hGCs and their ratios in captive-bred and wild-caught Iberian lynx. Measurements of cortisol, cortisone, DHEA, as well as cortisol-cortisone and cortisol-DHEA ratios calculated from HPLC-MS/MS results, in hair samples of captive-bred (*n* = 6) and wild-caught (*n* = 6) Iberian lynxes

Adult males presented significantly higher glucocorticoid levels measured by cortisol-3CMO-EIA compared to all juveniles and higher cortisone EIA values compared to adult females and juveniles. Glucocorticoid levels can fluctuate in mammal species depending on age, sex and season. Based on our previous work with the Egyptian mongoose (Azevedo *et al*., 2019), we expected hGC measured with cortisol-3CMO-EIA to be higher in young juveniles and in males, when compared to other age-sex groups. The conflicting results we observe for young Iberian lynx are probably due to the fact that all juveniles in our sample are older than 6 months of age and beyond the post-weaning phase where increased levels of hGC were observed in the mongoose. However, the cortisol-3CMO-EIA measurements also conflict with the HPLC-MS/MS measurements, where we found higher cortisone in males compared to females, but the opposite for cortisol. The cross-reaction of the cortisol-3CMO-EIA with cortisone and the proven presence of cortisone in lynx hair (see HPLC immunogram in Fig. 4) partially explain this conflict by contributing to an overestimation of EIA cortisol measurements in females and an underestimation in males. A comparison of the HPLC-MS/MS and EIA results for 12 Iberian lynx hair samples shows a 2-fold overestimation with the cortisol-3CMO- EIA, whose values were similar and significantly correlated to the sum of cortisone and cortisol obtained by HPLC-MS/MS. This is yet another example of where choosing the wrong assay can lead to erroneous results (Jewgenow *et al*., 2020).

DHEA was the most abundant steroid in Iberian lynx hair. This androgen with anti-glucocorticoid effects is produced in the adrenal glands and the brain (Maninger *et al*., 2009) and has been proposed as an indicator of allostatic load in humans (Mcewen, 2004). The mechanisms by which DHEA exerts its anti-glucocorticoid effects are not fully known, but it has been shown to decrease the expression of 11β-HSD-1 and increase the expression and activity of 11β-HSD-2 in mice, both *in vivo* and in cell cultures (Maninger *et al*., 2009). In human skin samples, its metabolites 7α- and 7β-hydroxy-DHEA competitively inhibit cortisone binding to 11β-HSD-1 and consequently its conversion to cortisol (Hennebert *et al*., 2007). In a similar approach as the one used for cortisone, several studies in humans showed serum and salivary cortisol-DHEA ratios to be more accurate predictors of depression than DHEA or cortisol levels alone (Maninger *et al*., 2009).

We calculated cortisol-cortisone and cortisol-DHEA ratios in Iberian lynx hair, and assessed their variation between animals of different origin and sex, in comparison to the values of the single hormones (Table 1, Fig. 6). While trends are noticeable using the single hormones, with apparently higher cortisol and lower cortisone concentrations in wild-caught Iberian lynxes of the captive breeding program compared to their captive-bred counterparts, these differences were not statistically significant. However, when combining cortisol and cortisone values into a cortisol-cortisone ratio, the differences between captive-bred and wild-caught animals become more evident (Fig. 6) and statistically significant (Table 1). More surprisingly, despite no apparent difference in DHEA levels between both groups, the cortisol-DHEA ratio was different between wild-caught and captive-bred Iberian lynxes, with the highest statistical significance of all variables tested. These findings are consistent with the hypothesis of a balanced mechanism using cortisone and cortisol interconversion through 11β-HSD enzymes regulated by DHEA. With regards to the above conclusions in relation to the animals’ origin, it is important to explain the opportunistic nature of our sample collection with five out of six wild-caught animals being females, one male and vice-versa in the captive-bred group. The wild-caught status is expected to be associated with higher stress levels in captivity, but the latter hypothesis is difficult to prove unequivocally in this study because of the potential sex bias. This again illustrates the difficulty in controlling for confounding factors when working with critically endangered species. Nevertheless, since the activity of 11β-HSD-1 and its regulation by DHEA metabolites has been demonstrated in human skin samples (Hennebert *et al*., 2007) and radiolabeled cortisol has been shown to be incorporated in hair as cortisone and other metabolites (Kapoor *et al*., 2018), it is difficult to dismiss the possibility that glucocorticoid levels found in Iberian lynx hair samples could be the end result of a similar system, possibly as part of a local HPA-axis analogue (Slominski *et al*., 2007). Unfortunately, most validation studies using repeated ACTH challenge (e.g. del Rosario *et al*., 2011; Terwissen *et al*., 2013) and known stressors (e.g. Davenport *et al*., 2006) have been performed using only cortisol assays. Further studies comparing the simultaneous variations in hair cortisol, cortisone and DHEA in response to repeated ACTH challenge and known stressors will be necessary to clarify this.

Finally, in order to explore the practical applicability of this concept, we compared the cortisol-3CMO-EIA and cortisone-EIA measurements for an Iberian lynx after a 24-day escape following a wildfire that affected the breeding center. In the 3 weeks following the escape-recapture event, there was an inversion of the ratio of the cortisol-3CMO-EIA to cortisone-EIA measurements, indicating a relative rise in cortisol concentration compared to cortisone (Fig. 3). Approximately 1 year later, the trend had inverted again, with higher cortisone-EIA measurements. Despite the limitation of the cortisol-3CMO-EIA, lacking specificity by partial cross reactivity with cortisone, the results do support the need for further research into the potential value of more than one steroid as an indicator of stress in this species.

Hormone measurement in hair using EIA can provide valuable tools for wildlife management and conservation. Limitations such as the lack of specificity due to antibody cross-reactivity need to be accounted for by prior cross-validation by HPLC and HPLC-MS/MS, as we demonstrate for progesterone and cortisol. Nevertheless, a more comprehensive approach that accounts for biologically relevant local metabolic pathways might be necessary to achieve the full potential of hair steroid measurement, as is becoming increasingly evident in the case of hGCs.

## Supplementary Material

Supplementary_coaa075
