# Supplementary material for: Sex steroids and glucocorticoid ratios in Iberian lynx hair
Source: Conserv Physiol. 2020 Aug 17;8(1):coaa075. doi: 10.1093/conphys/coaa075 (PMC7439579; doi:10.1093/conphys/coaa075)
Supplement: Supplementary_coaa075 [file supplementary_coaa075.docx]

**Supplementary data.** Table 1 Interassay and intraassay CV (%) of lower and higher quality control samples (concentration in pg/ml) for each assay. Linear range of the respective assays is also presented in brackets.

| **EIA** | **Interassay CV** | | **Intraassay CV** | |
| --- | --- | --- | --- | --- |
| **Cortisol-3-CMO**  (100–1000 pg/ml) | 240 pg/ml  6.2% (n = 4) | 550 pg/ml  6.9% (n = 4) | 255 pg/ml  8.4% (n = 16) | 690 pg/ml  9.5% (n = 16) |
| **Cortisone**[*****](#TF1)  (200–40 000 pg/ml) | 257.0 pg/ml  7.1% (n = 3) | 2925.5 pg/ml  3.9% (n = 3) | 14.1 pg/ml[*](#TF1)  7.4% (n = 20) | 349.2 pg/ml[*](#TF1) 6.2% (n = 20) |
| **Progesterone**  (125–1000 pg/ml) | 145 pg/ml  15.3% (n = 3) | 365 pg/ml  11.3% (n = 3) | 170 pg/ml  6.1% (n = 15) | 420 pg/ml  3.6% (n = 15) |
| **Testosterone-3-CMO**  (50–550 pg/ml) | 250 pg/ml  6.7% (n = 3) | 680 pg/ml[**](#TF2)  11.1% (n = 3) | 240 pg/ml  6.8% (n = 16) | 750 pg/ml[**](#TF2)  5.1% (n = 16) |

*According to the provider Arbor Assays, An Arbor, USA.

**QC was pre-diluted to fit linear range

**Supplementary data. Table 2** Steroid concentrations measured by EIA with two extraction methods, using pulverized and whole hairs.

| **Animal ID** | **Sex** | **Glucocorticoids (pg/mg)** | | **Progesterone (pg/mg)** | | **Testosterone (pg/mg)** | |
| --- | --- | --- | --- | --- | --- | --- | --- |
| **Method** | | **Pulverized** (n = 3) | **Whole** (n = 2) | **Pulverized** (n = 3) | **Whole**  (n = 2) | **Pulverized** (n = 3) | **Whole** (n = 2) |
| **IL87** | **Male** | 51.39 ± 8.35 | 52.85 ± 1.63 | 30.43 ± 4.26 | 16.81 ± 0.05 | 15.88 ± 2.82 | 7.98 ± 0.10 |
| **IL93** | **Male** | n.a. | 45.67 ± 6.49 | n.a. | 7.93 ± 2.26 | n.a. | 5.90 ± 0.03 |
| **IL97** | **Male** | 54.93 ± 3.70 | 47.04 ± 3.79 | 19.65 ± 0.19 | 6.97 ± 0.24 | 11.22 ± 0.86 | 4.26 ± 0.05 |
| **IL132** | **Female** | 43.82 ± 1.55 | 44.90 ± 0.71 | 75.41 ± 3.10 | 59.04 ± 13.78 | 13.55 ± 1.00 | 6.76 ± 1.08 |
| **IL138** | **Female** | 59.69 ± 7.95 | 48.82 ± 1.55 | 86.48 ± 8.08 | 120.38 ± 5.81 | 16.58 ± 2.01 | 10.92 ± 0.04 |
| **IL141** | **Female** | 45.41 ± 7.07 | 38.07 ± 7.40 | 98.02 ± 8.63 | 91.29 ± 20.89 | 14.28 ± 0.46 | 7.83 ± 0.71 |
| **Averaged all** | | 51.05 ± 8.15 | 46.23 ± 5.69 | 62.00 ± 32.66 | 50.40 ± 46.31 | 14.30 ± 2.41 | 7.27 ± 2.18 |
| **Averaged males** | | 53.16 ± 6.09 | 48.52 ± 4.84 | 25.04 ± 6.49 | 10.57 ± 4.96 | 13.55 ± 3.16 | 6.05 ± 1.67 |
| **Averaged females** | | 49.64 ± 9.35 | 43.93 ± 5.93 | 86.64 ± 11.54 | 90.24 ± 29.75 | 14.80 ± 1.79 | 8.50 ± 2.02 |

**Supplementary data. Table 3** Averaged coefficients of variation (CVs; %) (± SD) on 9 duplicates of the pulverized hair method and 10 duplicates of the whole hair method with and without an additional sample homogenization step.

| **Coefficients of variation (CV%** **±** **SD)** | **Without extra preparation step** | | **With extra preparation step** | |
| --- | --- | --- | --- | --- |
|  | **Whole hair**  **(n** **=** **10)** | **Pulverized**  **(n** **=** **10)** | **Whole hair**  **(n** **=** **6)** | **Pulverized**  **(n** **=** **5)** |
| **Cortisol-3-CMO-EIA** | 40.70 ± 24.64 | 42.64 ± 25.62 | 8.26 ± 7.18 | 11.57 ± 4.89 |
| **Progesterone-3-CMO-EIA** | 50.45 ± 52.26 | 50.05 ± 34.73 | 13.87 ± 12.33 | 7.45 ± 5.03 |
| **Testosterone-3-CMO-EIA** | 26.51 ± 16.26 | 24.92 ± 13.24 | 4.73 ± 6.45 | 9.63 ± 5.51 |

The extra step consisted in cutting the available hairs in 5 mm pieces and mixing the prior to weighing of 20 mg hair for extraction. An improvement in repeatability is noticeable with the addition of the extra step.

**Supplementary data. Table 4** Hair steroid hormone measurements using EIA

|  | **Cortisol-3CMO-EIA**  **(pg/mg)** | **Cortisone-EIA (pg/mg)** | **Progesterone-3CMO-EIA (pg/mg)** | **Testosterone-3CMO-EIA (pg/mg)** |
| --- | --- | --- | --- | --- |
| **Adult males** | 50.2 ± 9.9  (n = 8) | 45.9 ± 18.9  (n = 5) | 18.1 ± 6.9  (n = 8) | 10.8 ± 3.2  (n = 8) |
| **Adult females** | 42.6 ± 7.5  (n = 12) | 30.3 ± 5.4  (n = 9) | 85.2 ± 17.9  (n = 12) | 13.6 ± 2.9  (n = 12) |
| **Juvenile males** | 36.7 ± 6.6  (n = 8) | 23.0 ± 7.8  (n = 7) | 12.1 ± 8.3  (n = 8) | 10.5 ± 6.1  (n = 8) |
| **Juvenile females** | 38.4 ± 7.4  (n = 9) | 24.4 ± 6.3  (n = 8) | 9.6 ± 3.2  (n = 9) | 9.1 ± 3.6  (n = 9) |

Summary of mean ± SD for measurements using EIA targeting cortisol, cortisone, progesterone and testosterone in Iberian lynx hair samples grouped by age and sex. Our corticosterone EIA failed to detect corticosterone in all samples and is therefore omitted from the table.

**Supplementary data.** **Table 5** Effects of behavioral and management variables on cortisol-3CMO-EIA measurements in a group of Iberian lynx females.

| **Variable** | **Categories/Range** | **Result** | **Statistical method** |
| --- | --- | --- | --- |
| Breeding centre | SI, LO, EA | χ2(3) = 0.625, p = 0.89 | Kruskall-Wallis rank sum test |
| Origin | captive-bred, wild-caught | U = 77, p = 0.525 | Mann-Whitney U-test |
| Enclosure area (m^2^) | 200–1250 | rs(12) = −0.06, p = 0.801 | Spearman’s correlation |
| Days since last capture | 42–734 | rs(12) = 0.04, p = 0.893 | Spearman’s correlation |
| Total lifetime captures | 0–17 | rs(13) = −0.07, p = 0.816 | Spearman’s correlation |
| Days since last transfer | 343–4478 | rs(11) = −0.21, p = 0.494 | Spearman’s correlation |
| Total lifetime transfers | 0–5 | rs(13) = 0.02, p = 0.943 | Spearman’s correlation |
| Days since enclosure change | 4–627 | rs(13) = −0.05, p = 0.864 | Spearman’s correlation |
| Lifetime enclosure changes | 1–41 | rs(13) = −0.07, p = 0.805 | Spearman’s correlation |
| Repetitive behaviour (%) year | 1–17 | rs(10) = −0.16, p = 0.609 | Spearman’s correlation |
| Repetitive behaviour (%) month | 3–22 | rs(9) = −0.24, p = 0.474 | Spearman’s correlation |

The table summarizes the results of non-parametric tests for differences in cortisol-3CMO-EIA measurements in hair with behavioral (frequency of repetitive behavior in the month and year prior to hair sample) and management variables (breeding centre where the animal is held, origin, enclosure area and days since last- and total lifetime captures, institutional transfers and enclosure changes). No significant relation was detected between any of the behavioral and management variables and EIA measurements.

**Supplementary data. Table 6** Steroid hormone concentrations (by EIA) in proximal and distal hair segments

|  | **Cortisol-3CMO-EIA (pg/mg)** | **Cortisone-EIA (pg/mg)** | **Progesterone-3CMO-EIA (pg/mg)** |  | **Progesterone-3CMO-EIA (pg/mg)*** | **Testosterone-3CMO-EIA (pg/mg)** |
| --- | --- | --- | --- | --- | --- | --- |
| **Proximal** | 39.95 ± 6.34 | 35.30 ± 10.97 | 52.42 ± 41.95 |  | 52.42 ± 41.95 | 9.40 ± 2.52 |
| **Distal** | 44.27 ± 8.99 | 39.87 ± 19.65 | 389.65 ± 1057.09 |  | 55.54 ± 36.01 | 14.85 ± 4.36 |
| **Correlation coefficient** | 0.73 | 0.76 | 0.42 |  | 0.99 | 0.87 |
| **p-value** | 0.017 | 0.016 | 0.225 |  | 4.973e-08 | 0.001 |

Summary of mean ± SD, correlation coefficients and p-values for comparison of steroid concentrations in proximal and distal hair segments of Iberian lynx hair samples, measured by EIA. *Progesterone values after removal of one outlier value of distal hair segment for Dama (3396.64 pg/mg). The outlier is an order of magnitude higher than the next highest value and over 60 times higher than the mean of all progesterone values; we therefore considered it unlikely to be biologically plausible.

**Supplementary data. Table 7** Hair steroid concentrations determined by comparative HPLC-MS/MS

|  | **cortiso**l  (pg/mg) | **cortisone** (pg/mg) | **hGC** (pg/mg) | **T** (pg/mg) | **P4**  (pg/mg) | **DHEA** (pg/mg) | **CC** (pg/mg) | **Cortisol-EIA** (pg/mg) | **P4-EIA**  (pg/mg) |
| --- | --- | --- | --- | --- | --- | --- | --- | --- | --- |
| **Mean** | 13.35 | 24.82 | 38.16 | 2.36 | 9.21 | 33.73 | 0.00 | 37.27 | 32.60 |
| **± SD** | ± 7.80 | ± 9.58 | ± 12.27 | ± 2.04 | ± 5.94 | ± 14.14 | n.d. | ± 13.24 | ± 34.2 |

HPLC-MS/MS values of cortisol, cortisone, sum of cortisol and cortisone (hGC), testosterone (T), progesterone (P4), DHEA and corticosterone (CC) in a total of 12 Iberian lynx hair samples. Cortisol (cortisol-EIA) and progesterone (P4-EIA) concentrations determined by the respective EIA from the same 12 samples were included for comparison.
